# Hepatitis C virus (HCV) Infection Rate among Seronegative Hemodialysis Patients Screened by Two Methods; HCV Core Antigen and Polymerase Chain Reaction

**DOI:** 10.5812/hepatmon.9147

**Published:** 2013-05-30

**Authors:** Maryam Moini, Mazyar Ziyaeyan, Shapoor Aghaei, Mohammad Mahdi Sagheb, Seyed Alireza Taghavi, Mahsa Moeini, Marzieh Jamalidoust, Laleh Hamidpour

**Affiliations:** 1 Gastroenterohepatology Research Center, Shiraz University of Medical Sciences, Shiraz, IR Iran; 2 Professor Alborzi Clinical Microbiology Research Center, Shiraz University of Medical Sciences, Shiraz, IR Iran; 3 Department of Internal Medicine, Yasouj University of Medical Sciences, Yasouj, IR Iran; 4 Nephrourology Research Center, Shiraz University of Medical Sciences, Shiraz, IR Iran

**Keywords:** Hepatitis C Virus, Core Antigen, Hemodialysis, Polymerase Chain Reaction

## Abstract

**Background:**

End-stage renal disease patients on chronic hemodialysis are among high risk groups for hepatitis C virus (HCV) infection for whom routine HCV screening is recommended. Anti-HCV antibody (ab) testing may not be reliable to detect all infected cases because of the blunted ab response due to depressed immune state in these patients. Using a more reliable, cost-effective and non-complex HCV screening test may be necessary in this group of patients for case finding and management, and also for prevention of infection spread.

**Objectives:**

The aim of this study was to find the prevalence of HCV infection in HCV ab negative hemodialysis patients by Real time PCR and total HCV core antigen (ag) test and comparing the results of the two tests.

**Patients and Methods:**

From a single hemodialysis center, 181 anti- HCV ab negative patients were screened by total HCV core ag using an ELISA kit. Real time PCR was used for determination of the virus and viral load quantity.

**Results:**

Among the 181 anti-HCV ab negative patients, 13 (7.2%) were positive for HCV core ag and 11 (6%) had detectable HCV RNA with a range of 40-336543 IU/ml by PCR. The two tests had a high measurement agreement (Kappa=0.82, P<0.001). Of the 13 patients with positive HCV core ag test results, 3 were negative for HCV RNA. Considering real time PCR for HCV RNA as the gold standard for HCV infection determination in this patient population, HCV core ag assay yielded a sensitivity of 90.9%, specificity of 98.2%, positive predictive value of 76.9% and negative predictive value of 99.4%.

**Discussion:**

The rate of HCV infection among HCV ab negative hemodialysis patients was high. HCV core ag testing could be used as a sensitive method for HCV infection screening in this group of patients.

## 1. Background

Patients on chronic hemodialysis are at increased risk of hepatitis C virus (HCV) infection (1). Chronic kidney disease patients ever treated by hemodialysis are recommended to be screened for HCV infection (2). The prevalence of infection has decreased but still remains high despite the decreased risk of transfusion related to viral transmission by effective blood screening (3). The rate of HCV infection in hemodialysis patients varies among different countries. The usual screening tests used for HCV infection are based on antibody (ab) detection using enzyme immunoassay (EIA). These tests are easily usable with a relatively low cost; however, despite the improved sensitivity of higher generations, there are some limitations for their use, especially for this particular population of patients. The window period between acute infection and ab production may be more prolonged in end-stage renal disease (ESRD) patients due to the immunodeficiency state (4). Another weak point for ab detection tests is their inability to differentiate the concurrent infection from the resolved past infection. This is while methods for direct viral detection such as polymerase chain reaction (PCR) and nucleic acid amplification technology (NAT) have their own limitations for mass screening, including their expense, high technical skill requirement and long incubation time (for NAT). There are also reports on the value of HCV core antigen (ag) assay for infection detection and its special application for hemodialysis patients in the literature (5-9).

In Iran, the rate of HCV infection in hemodialysis patients has been reported to be about 13.7% by HCV ab assay (10). However, determination of the prevalence of infection in hemodialysis units would not be real without considering the infection rate in the HCV ab negative group. In the present study anti-HCV negative hemodialysis patients in one of the largest hemodialysis centers in Iran were screened using two methods: PCR and HCV core ag EIA with the purpose of detecting the infection rate in this population of patients and also to compare the ability of HCV core ag test for infection detection with PCR as a screening tool.

## 2. Objectives

The present study was designed to determine the rate of HCV infection in a group of hemodialysis patients who are usually underdiagnosed due to their negative HCV ab results. In the purpose to suggest a simple reliable screening test, HCV core ag test results were compared with Real time PCR results in these patients.

## 3. Patients and Methods

This study was done during screening for viral hepatitis which is routinely done every 6 months at Haj Ebrahimi Hemodialysis Center of Shiraz University of Medical Sciences in March 2009. All patients on chronic hemodialysis with durations of more than 6 months were included in this study. Patients’ charts and documents were reviewed by the investigator. The demographic data and characteristics for each patient were written down in a data form in addition to other data, including, the cause of renal disease and duration of hemodialysis. Every patient was also interviewed by the investigator and questioned about the other possible risk factors for viral hepatitis; history of drug use, blood transfusion and previous solid organ transplantation. All patients were specifically asked about the history of acute hepatitis, jaundice or liver disease at any time during their life. Patients who were known to be seropositive for hepatitis C ab and/or hepatitis B surface ag (HBs Ag) were excluded from the study. At the beginning of the hemodialysis session, 10 ml of blood was collected in a tube by venipuncture from each patient, immediately centrifuged for serum separation and transferred to the research lab. All serum samples were stored at -700 °C for further testing. All 185 samples were tested for HBs Ag (Diapro, Milano, Italy) and anti-HCV ab using third generation Enzyme-linked immunosorbent assay (ELISA) kit (Diapro, Milano, Italy). Samples which were positive for anti-HCV ab (4 samples) were excluded from further testing. None of the samples was positive for HBs Ag. All 181 anti-HCV ab negative samples were tested for HCV RNA and HCV core ag.

### 3.1. HCV Core Ag Test

For the HCV core ag assay, a 3-stage test was done using an ELISA kit (Johnson and Johnson, Ortho Clinical Diagnostic, New Jersey, USA) according to the instructions provided by the manufacturer. This test was carried out in microwells coated with monoclonal antibodies that recognize HCV core ag. The results were interpreted as positive or negative according to the standard curve.

### 3.2. RNA Extraction (Nucleic Acid Extraction)

Total RNA extraction was performed using the handmade RNA extraction method as follows:

Briefly, 200 µl of the serum was mixed with 800 µl of the RNX reagent and 200 µl of chloroform was added. Following centrifugation at 800 g, the upper layer containing RNA was precipitated by ethanol and the pellets were washed and dissolved in 30 µl nuclease free water and stored at –70˚C for a maximum duration of 3 days to be used for reverse transcriptase PCR (RT–PCR) for HCV virus.

### 3.3. Reverse Transcriptase Real Time PCR

To detect HCV RNA, Taqman Real Time PCR was performed with the Hepatitis C Virus quantification Kit (Genome Diagnostics, Australia) according to the manufacturer’s instructions and by the Rotor Gene 2000/3000 Real-time PCR machine (Applied Biosystem Sequence Detector 7500 machine, USA). The threshold cycle values from the clinical samples were plotted on the standard curve and the numbers of copies were automatically calculated. For each run, positive and negative controls were included.

### 3.4. Statistical Analysis

Statistical analysis was performed using the Statistical Package for the Social Sciences (SPSS, Chicago, IL, USA)) release 16 for Windows. Non-parametric Mann–Whitney test was used for comparing the means of age, duration of hemodialysis, transfusion history, previous kidney transplantation and history of jaundice. A two-sided P value ≤ 0.05 was considered significant. The sensitivity and specificity of the HCV core Ag test were calculated according to the standard formula. Cross tabulation was done for the positive and negative results of HCV core Ag and RT-PCR tests to measure the agreement for the two tests. Prior approval for the experimental procedure was obtained from the ethical committee of Shiraz University of Medical Sciences, Shiraz, Iran. All volunteers were informed about the method of the study and potential risks. Every subject signed the informed consent and then enrolled in the study.

## 4. Results

One hundred eighty one patients with ESRD on chronic hemodialysis with mean age of 52.86 ± 18.33 (mean ± SD) were enrolled in this study. Seven pediatric patients were also included in the study group. The majority of patients were male (65.2% vs. 34.8%). Duration of hemodialysis was 20.32 ± 7.41 (mean ± SD) months on average with a range of 11 to 100 months. Ninety three (51.4%) patients had a history of blood transfusion at least once. Fifteen patients had previously received kidney transplantation, complicated by graft dysfunction. None of the patients had a history of intravenous substance abuse. Patients’ demographic data and characteristics have been presented in Table 1.

**Table 1.** Demographic Data and Characteristics of all Hemodialysis Patients Included in This Study

	Data
**Age, y, Mean, (range)**	52.7 (7-86)
**Gender, male, %**	65.2
**Duration of Hemodialysis, min, Mean (range)**	20.3 (11-100)
**History of Transfusion, No. (%)**	93 (51.4)
**History of Previous Renal Transplant, No. (%)**	15 (8.3)
**History of Jaundice, No. (%)**	1 (0.6)
**Etiology of ESRD**	
**Diabetes Mellitus, %**	27.6
**Hypertension, %**	25.4
**Failed transplanted kidney, %**	8.3
**Glomerulonephritis, %**	5.0
**Polycystic kidney disease, %**	3.9
**Others ** **^a^** **, %**	11.6
**Unknown, %**	18.2

^a^ Other etiologies include: drug induced nephropathy, unresolved acute kidney injury, pyelonephritis and urinary stone disease.

Of all the 181 anti-HCV Ab negative serum samples, 11 (6%) turned out positive for HCV RNA by the RT-PCR test. Patients had an average age of 57.63 ± 26.06 (mean ± SD) and sex distribution of 5 males and 6 females. The range of HCV RNA level varied from 40 to 336543 IU/ml. This group of patients had been on hemodialysis with a mean duration of 21.27 ± 4.67 (mean ± SD) months. Thirteen (7.2%) out of the 181 patients with an average age of 59 ± 24.09 (mean ± SD) were positive for total HCV core ag. There were 6 males and 7 females. Duration of hemodialysis was 20.38 ± 4.75 (mean ± SD) months on average, in this group of patients. There was no history of jaundice. Nine patients had a history of blood transfusion but none of them had previous kidney transplantation (Table 2). The measured agreement for the two tests was high (Kappa = 0.82, P < 0.001). Three patients with positive serology for HCV core ag were negative for HCV RNA and only one patient with a positive result for HCV PCR had a negative serology for HCV core ag. This patient had a low viral load by the PCR method (100 IU/ml). Out of 181 screened patients, 167 were negative for both HCV RNA and HCV core ag (Table 2). As a result, HCV core ag assay yielded a sensitivity of 90.9% and specificity of 98.2%, regarding RT- PCR for HCV RNA as the gold standard test for infection detection in this patient population. The positive predictive value for HVC core test was 76.9% in this study and the negative predictive value was calculated as 99.4%. The group of patients who were positive for HCV either with HCV core EIA or RT-PCR, were not different from patients who were negative in mean age, duration of hemodialysis, history of transfusion, previous transplant history or history of jaundice (P < 0.05).

**Table 2.** Comparison Between RT-PCR and EIA Results of all Hemodialysis Patients Included in This Study

	RT-PCR		
	Positive	Negative	Total, No. (%)
**HCV-Core Ag**			
Positive	10	3	13 (7.2)
Negative	1	167	168
**Total, No. (%)**	11 (6)	170	181

## 5. Discussion

HCV infection has a negative impact on the survival of hemodialysis patients, attributed mostly to HCV-related liver disease and its complications (11). There is also concern about some adverse outcomes after kidney transplantation in HCV-infected patients. Reduced long term patient and graft survival have been observed in HCV-infected kidney recipients (12, 13). Liver related complications such as cirrhosis and hepatocellular carcinoma are among the major causes of increased mortality in this population of patients, and in patients who are the candidates for antiviral treatment, increased risk of renal allograft dysfunction would be a major issue for safety of interferon based treatment regimens, interfering with optimal management of post kidney transplant HCV infection (14). Treatment of Hepatitis C infected ESRD patients before kidney transplantation would be a reasonable strategy to decrease post-transplant liver related complications and prevent the potential risk of kidney rejection during HCV treatment in post-transplant course (15). Therefore, through evaluation of the patient’s candidates for kidney transplantation, using sensitive tests is necessary to detect any evidence of hepatitis C infection in the pre transplant era. Prompt diagnosis of HCV infection in patients on chronic hemodialysis is important not only to offer the appropriate treatment for those who require it, but also to decrease the rate of infection transmission in hemodialysis units. The risk of HCV transmission from infected patients may be significant, even in the window period before ab production which was historically notified first after the outbreak of acute hepatitis C in the recipients of ab screened intravenous immunoglobulin in 1994 (16). The use of sensitive third generation ab detection assays has not resulted complete omission of the risk of HCV transmission from blood products donated by recently infected people. In patients with ESRD the prolonged window period and the risk of infection spread through hemodialysis devices are among other reasons to look for a simple, reliable diagnostic method for HCV infection even before anti-HCV ab production, in this population of patients, as a critical step for the control of infection spread in hemodialysis units.

Hepatitis C infection rate in hemodialysis units may be related to the infection’s prevalence in the general population of each region. Iran is among the low endemic areas for hepatitis C infection (17). The prevalence of HCV infection in a large population based study was reported to be 0.5% by Merat et al. (18) and the prevalence rate in Iranian hemodialysis patients was reported to be 13.7% by EIA HCV ab tests and 7.6% by immunoblot/PCR assays in a systematic review by Alavian et al. (10) which is quite low compared with many other countries in the Middle East region and also in Asia, Europe and America. The rate of HCV ab negative viremia detected by PCR in hemodialysis patients was reported to be 0-12% globally (19). A false negative rate of 17.9% has been detected for HCV ab among Egyptian hemodialysis patients (20) and false negative rates of 17.1% and 25.7% were reported from Saudi Arabia for ELISA and RIBA tests, respectively (21). In Iran most of the published reports on HCV prevalence rate in hemodialysis patients are based on HCV ab detection tests as the screening tool. There are a limited number of studies that have used PCR for infection detection, among which most have used PCR only for confirmation of infection in cases with positive samples for HCV ab, not as a screening test in all of the studied patients. Our study was done at the Haj Ebrahimi Hemodialysis Center, one of the largest hemodialysis centers in Iran. Despite other studies on the prevalence of HCV infection in Iranian hemodialysis patients, the screening was done only on patients with negative serums for HCV ab, using PCR and also HCV core ag test for direct viral or viral particles detection. Our results revealed that a significant percentage of seronegative hemodialysis patients had evidence of HCV infection by these two tests, which is in contrast to the Makhlough et al. survey which revealed positive HCV PCR results only in HCV ab positive samples while screening all hemodialysis patients regardless of their ab status (22).

The high false negative reports for HCV ab tests in hemodialysis patients according to the result of the current and other similar studies signify that a considerable number of HCV infected patients who would be potential sources for infection spread throughout the units may escape detection by relying only on these tests as the screening tools in hemodialysis units. For a more precise screening in this population of patients, tests that directly measure the virus particles are preferred. RT-PCR is the gold standard method for the diagnosis of HCV infection, allowing serum HCV RNA determination; however, obstacles such as technical difficulties, unavailability and expenses may prevent it from being used as a screening test on a large scale of patients on a regular basis. HCV core ag assay is the other test option to detect HCV ab negative infected cases. HCV core protein is a structural protein whose primary function is the formation of viral nucleocapsid. This protein particle is immunogenic and plays a role in host cellular and humoral responses. Core protein is the only HCV ag that can be detected by immunologic assays (23). The immunoassay for detection of HCV core ag was developed in the 1990s (24-26) and its utility for detection of HCV infection in immunocompetent individuals has been addressed by several other studies (27-29). This highly specific assay has been evaluated for detection of HCV infection in blood donors who tested negative for HCV ab in previous studies and was shown to be quite valuable for this purpose (27, 30, 31). The sensitivity and specificity of the test for HCV core ag for infection detection in a population of hemodialysis patients were reported to be 84% and 89% respectively by Bouzgarrou et al (5). They also noted the ability of the test for early detection of acute hepatitis C infection before ab detection in 3 of their patients (5). In a cohort study performed on hemodialysis patients, Fabrizi et al. reported a significant correlation between HCV core ag concentration and the level of HCV RNA measured by RT-PCR (6). Medhi et al. found HCV core ag test to be an accurate assay for early detection of infection in another study on hemodialysis patients (7) and even Cavoli et al. reported a positive predictive value of 100% for the test in this population of patients (32). Despite the promising reports on the ability of HCV core ag immunoassay for early detection of infection in hemodialysis patients, Reddy et al. reported a lower sensitivity rate (60%) for the test according to their study and stated that a single negative HCV core ag test may not be reliable enough to exclude early HCV infection (9). In the current study, which was done only on those hemodialysis patients who were serologically negative for HCV ab, the results of HCV core ag test were well correlated with the results from the HCV PCR test. The test especially gained a high negative predictive value (99.4%) that shows its appropriateness as a test for exclusion of infection.

In conclusion, in this study we note that the number of HCV infected hemodialysis patients with negative serology for HCV ab is significant. Comparing immunoassay method (EIA) to detect HCV core ag with RT-PCR for detection of HCV RNA as a gold standard test, HCV core ag detection test could be used as a screening test in HCV ab negative patients on hemodialysis based on its accuracy, simplicity and low expense.
